# Review of Noninvasive or Minimally Invasive Deep Brain Stimulation

**DOI:** 10.3389/fnbeh.2021.820017

**Published:** 2022-01-18

**Authors:** Xiaodong Liu, Fang Qiu, Lijuan Hou, Xiaohui Wang

**Affiliations:** ^1^School of Kinesiology, Shanghai University of Sport, Shanghai, China; ^2^Department of Exercise Physiology, Beijing Sport University, Beijing, China; ^3^College of Physical Education and Sports, Beijing Normal University, Beijing, China

**Keywords:** deep brain stimulation, focused ultrasound, temporal interference, nanoparticle, neuromodulation

## Abstract

Brain stimulation is a critical technique in neuroscience research and clinical application. Traditional transcranial brain stimulation techniques, such as transcranial magnetic stimulation (TMS), transcranial direct current stimulation (tDCS), and deep brain stimulation (DBS) have been widely investigated in neuroscience for decades. However, TMS and tDCS have poor spatial resolution and penetration depth, and DBS requires electrode implantation in deep brain structures. These disadvantages have limited the clinical applications of these techniques. Owing to developments in science and technology, substantial advances in noninvasive and precise deep stimulation have been achieved by neuromodulation studies. Second-generation brain stimulation techniques that mainly rely on acoustic, electronic, optical, and magnetic signals, such as focused ultrasound, temporal interference, near-infrared optogenetic, and nanomaterial-enabled magnetic stimulation, offer great prospects for neuromodulation. This review summarized the mechanisms, development, applications, and strengths of these techniques and the prospects and challenges in their development. We believe that these second-generation brain stimulation techniques pave the way for brain disorder therapy.

## Introduction

Neuromodulation has attracted considerable attention worldwide for its value in treating neurodegenerative diseases and increasing human performance, and many countries have increased investment and built their brain projects to accelerate the development process of neuromodulation. Brain stimulation, a part of the brain project, plays a crucial role in neuroscience research and clinical application and has an advantage over pharmacotherapy because of its fast, direct, and focal effects. Brain stimulation can alter neuronal activities through the delivery of a stimulus to targeted brain areas, thus alleviating brain disorders or enhancing brain functions. Brain stimulation is a multidisciplinary research field, which involves neurophysiology, bioengineering, and material and computer science (Tatti et al., 2016; Antal et al., 2017).

The most commonly employed transcranial brain stimulation techniques include transcranial magnetic stimulation (TMS), transcranial direct current stimulation (tDCS), and deep brain stimulation (DBS; Adair et al., 2020). TMS and tDCS represent major noninvasive neurostimulation techniques, which have been widely used in clinical research for decades (Begemann et al., 2020). However, the effects of noninvasive brain stimulation through TMS and tDCS on neurons vary and are difficult to assess. Furthermore, magnetic and electric signals show absorption and scattering properties within brain tissues, limiting spatial resolution and penetration depth (Woods et al., 2016; Airan, 2017). DBS is an invasive neuromodulation that requires the implantation of stimulating electrodes to deep brain structures; these electrodes can precisely target deep brain nuclei through the direct control of brain circuit dynamics (Parker et al., 2020). DBS has been widely used in alleviating neurological disorders, such as motor and cognitive dysfunctions, which cannot be alleviated by traditional therapies (Lozano and Lipsman, 2013). In particular, DBS of the subthalamic nucleus (STN) is one of the most effective treatments for Parkinson’s disease (Habets et al., 2018). However, DBS requires chronic implantation deep in the brain, which may eventually suffer from bleeding and infection (Kim et al., 2016). The above techniques lack cell specificity and thus have limited efficacy (Dayan et al., 2013). Thus, noninvasive and precisely deep stimulation represents a major breakthrough in neuroscience.

For this problem, exploring novel brain modulation techniques that satisfy the requirements of research and clinical application should be explored. Ideal brain stimulation targeting deep brain regions should be noninvasive or minimally invasive and have a high spatiotemporal resolution and negligible inflammatory or complications in different animal models, including rodents, large mammals, non-human primates, and humans (Li et al., 2021). Great advances have been achieved by neuromodulation studies in the past decade, driven by improvements in methods and devices. In particular, second-generation brain stimulation techniques that mainly rely on acoustic, electronic, optical, and magnetic signals exhibit great promise for neuromodulation (Lewis, 2016; Lozano, 2017; Chen, 2019; Darrow, 2019). These novel techniques are aimed at surpassing the limitations of conventional brain stimulation approaches. Some current approaches are limited to laboratory research. Nevertheless, some methods have already been used in clinical applications. Here, we provide an overview of these techniques and outline the prospects and challenges in future development.

## Focused Ultrasound

**Figure 1:**
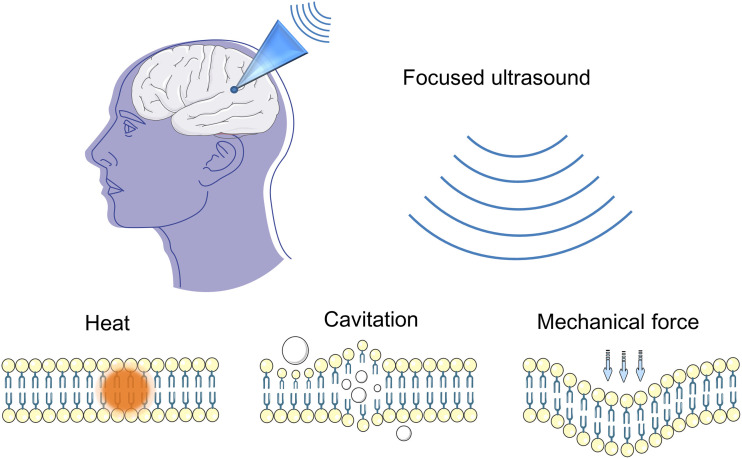
Focused ultrasound neuromodulation. The potential biophysical effects of ultrasonic neuromodulation.

Focused ultrasound (FUS) is a noninvasive neuromodulation technique with high spatial resolution and penetration depth (Figure 1; Fini and Tyler, 2017). FUS can deliver mechanical forces, penetrate biological tissues in small deep brain regions, and form a focal spot that can result in thermal and mechanical bioeffects (Kubanek, 2018; Blackmore et al., 2019). Ultrasound is a mechanical pressure wave with frequencies above the human audible range. As a propagating wave, ultrasound can alter neuronal activity by stimulating nerves and muscles (Harvey, 1929). Fry et al. (1958) first reported that ultrasound considerably affects brain activity, and they used high-intensity focused ultrasound (HIFU) for movement disorders and chronic pain (Fry, 1958). After decades of development in basic FUS technology, FUS especially Low-intensity ultrasound (LIFU) has burst a great breakthrough in scientific research and clinical treatment, continuously creating new possibilities in neuroscience (Rabut et al., 2020).

### Mechanisms of FUS

FUS has many interaction modes for tissues, and these modes depend on FUS parameters, including thermal, cavitation, and mechanical mechanisms. Ultrasound can be defined as high intensity (>1 W/cm^2^) or low intensity (<300 mW/cm^2^; Tufail et al., 2010). The biological effects of HIFU are mainly local heating effects. The heating effects homogenize tissues and denatured proteins (Ishibashi et al., 2010). LIFU has been reported to have a great number of effects on neuromodulation. LIFU can create minimal temperature elevation. Even a small variation in temperature affects ion channels and enzymatic and potential activities (Darrow, 2019). Most studies indicated that the neuromodulation of LIFU is due to nonthermal mechanical mechanisms. Mechanosensitive ion channels, which can respond to mechanical stimuli, may mediate neural response to FUS (Ye et al., 2018). Mechanical forces exerted by FUS induce membrane displacement and mediate conformational change in embedded ion channels (Kubanek, 2018). In addition, cavitation elicited by LIFU is considered a mechanism of neuromodulation. Cavitation produces microbubbles that cause the collapse of soft tissues, and shear stress temporarily alters tight junctions and increases the permeability of the blood–brain barrier (BBB; Chu et al., 2015; Kubanek, 2018).

### Development and Applications of FUS

FUS is valuable to neuroscience research and clinical applications. A large number of studies used FUS in different models, including neural tissues, rodents, non-human primates, and humans. In *in vitro* studies, FUS was first applied to brain modulation in the 1950s. It caused reversible suppression for sensory-evoked potential in the primary visual cortices of cats through the lateral geniculate nucleus (Fry et al., 1958). Mihran et al. (1996) determined whether or not the mechanical vibration of FUS applied to neural and cardiac cells can modify cellular excitability. Low-energy FUS increased conduction velocity and compound action potential in the excised sciatic nerves of a bullfrog (Tsui et al., 2005). In microelectrode arrays for observing the spatiotemporal dynamics of extracellular neuronal activities after FUS, local field potential spread across hippocampal sections (Suarez-Castellanos et al., 2021).

Following the initial discovery using FUS *in vitro*, animal behavioral effects and network activity changes have been investigated *in vivo* (King et al., 2013; Yu et al., 2016; Yuan et al., 2020). Tufail et al. (2010, 2011) reported that LIFU can promote sharp-wave ripple oscillations and trigger electromyogram (EMG) activities and forepaw and tail movements. Yuan et al. (2020) found that LIFU induces rapid hemodynamic responses at stimulation sites and demonstrated linear coupling relationships among cortical blood flow, local field potential, and EMG amplitude. Baek et al. (2018) revealed that LIFU generates motor-evoked potential (MEP) and enhances sensorimotor recovery in stoke mice and found that cerebellar LIFU leads to a symmetrical decrease in pathological neural activities and enhances recovery in stoke mice. Yoo et al. (2011) demonstrated that FUS can be applied to rabbit deep brain structures and neuronal activities can be activated or suppressed depending on FUS parameters. FUS effects in large animals were further investigated, and the results suggested that FUS-mediated brain stimulation can be precise, effective, and safe in ovine models (Yoon et al., 2019). Deffieux et al. (2013) examined awake macaque rhesus monkeys; they showed that LIFU significantly modulates antisaccade task latencies; and they demonstrated the feasibility of using LIFU in modulating high-level cognitive behavior. What is more, FUS transiently and reversibly changes brain activities in deep cortical and subcortical regions with high spatial resolution, and modulatory effects on active and resting neurons vary (Folloni et al., 2019; Yang et al., 2021).

Moreover, the molecular responses of FUS have been recently reported. Data has shown that LIFU can stimulate brain activities involved in the activation of voltage-gated sodium and calcium channels (Tyler et al., 2008). LIFU modulates the level of neurotransmitters, Min observed a significant increase of the extracellular levels of dopamine and serotonin (Min et al., 2011). Oh et al. (2019) found that ultrasound-induced neuromodulation is initiated by transient receptor potential A1 (TRPA1) in astrocytes; TRPA1 causes a release of glutamate; finally activates N-methyl-D-aspartic acid receptors in neighboring neurons. The expression levels of neurotrophic factors, such as brain-derived neurotrophic factor (BDNF), glial cell line-derived neurotrophic factor, and vascular endothelial growth factor, can be increased by LIFU in the rat models of Alzheimer’s disease (Lin et al., 2015). BDNF expression is upregulated through the activation of tropomyosin-related kinase B, phosphatidylinositol-3-kinase (PI3K), protein kinase B (Akt), and calmodulin kinase signaling pathways (Liu et al., 2017). FUS exposure suppresses epileptic activities in acute epilepsy rat models, and this effect seems to be mediated by the PI3K-Akt-mammalian target of rapamycin (mTOR) signaling pathway (Chen S.-G. et al., 2020).

In addition to the animal results, many researchers targeted human studies. FUS on the motor cortex transiently increases motor cortex excitability and decreases reaction time during visual motor tasks (Gibson et al., 2018; Fomenko et al., 2020). Legon et al. (2018) successfully combined electroencephalographic, computed tomography (CT), and magnetic resonance imaging (MRI) to assess the effects of FUS neuromodulation on humans. The study revealed that FUS shows perfect spatial precision and resolution when used in modulating human subcortical deep brain regions, such as the unilateral thalamus (Legon et al., 2018).

### Strengths and Challenges of FUS

FUS is a promising noninvasive deep brain neuromodulation approach with high spatial precision, resolution, and safety and can reversibly modulate brain activities in subcortical and deep cortical regions with millimetric range neurostimulation (Tufail et al., 2010; Deffieux et al., 2013). Portable, wearable, and array transducer FUS has been used in research, so as to better perform its function (Li et al., 2018, 2019). FUS devices are constantly developed to be more suitable for clinical practice. Using nanoparticles that specifically target drugs in specific brain areas have been used as mediators to improve the targetability of FUS (Ozdas et al., 2020; Hou et al., 2021). FUS has been used effectively and safely for neuromodulation in small animals, non-human primates, and humans (Legon et al., 2018; Folloni et al., 2019; Baek et al., 2020) and is compatible with MRI and CT imaging techniques, showing considerable potential as a neuromodulation method for disabling neurological disorders. Clinical trials using FUS have been conducted for the treatment of Alzheimer’s disease, Parkinson’s disease, epilepsy, and stroke (Meng et al., 2017; Fomenko et al., 2020).

Although a number of studies have shown that FUS is safe and effective, further prospective work is needed to elucidate parameters for safety and effectivity threshold. The short- and long-term effects of FUS need to be treated differently. Basic experiments should focus on illuminating the potential cellular, molecular, synaptic, and ionic mechanisms of FUS neuromodulation.

## Temporal Interference Stimulation

**Figure 2:**
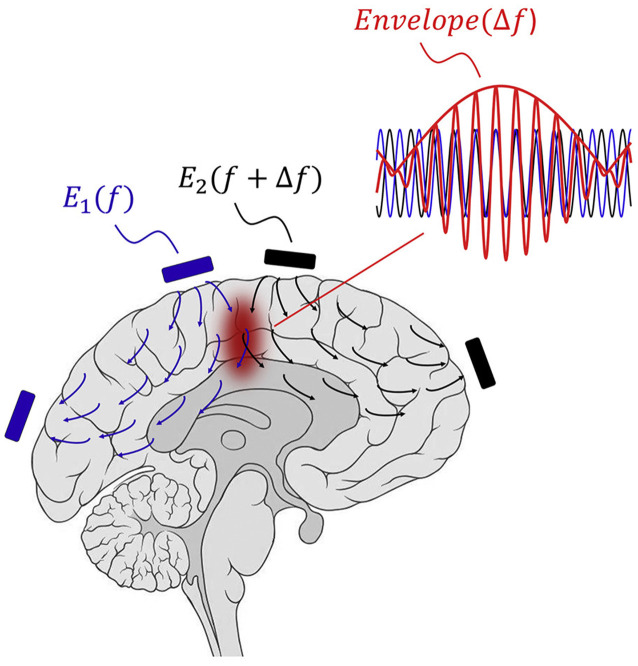
Concept of TI stimulation. The interference of two oscillating electric fields with slightly different frequencies (f1 = 2,000 Hz; f2 = 2,010 Hz; Δ*f* = 10 Hz) produces an envelope equal to Δf in current intersection regions (Copyright permission was obtained from the publisher; Grossman et al., 2017). TI, Temporal interference.

Temporal interference (TI) stimulation is a novel noninvasive transcranial electrical stimulation (TES) technique that can reach deep brain regions (Figure 2). It utilizes multigroup high frequency (≥1,000 Hz) and oscillating electric fields in modulating neural activities. In 1965, The TI concept was proposed by Russian scientists and applied to peripheral stimulation (Beatti et al., 2011; Guleyupoglu et al., 2013; Li et al., 2020). Then, Grossman et al. (2017) used TI stimulation in brain research in 2017. Since then, TI has attracted the attention of neuroscientists because it may achieve noninvasive deep brain stimulation. The position of TI stimulation can be changed by adjusting electrode location and current amplitude ratio. It can target deep brain regions and prevent the activation of the neighboring and overlying cortex. TI stimulation offers a means to precisely regulate subcortical structures.

### Mechanisms of TI Stimulation

TI stimulation consists of two sets of high frequency sinusoidal waveform currents with slightly different frequencies (*f*_1_ = 2,000 Hz; *f*_2_ = 2,010 Hz; Δ*f* = 10 Hz). Two high frequency oscillating electric fields interact and produce an envelope that is equal to Δf in current intersection regions. Owing to neural biophysical properties, neural membranes respond to low-frequency electrical signals, and high-frequency oscillation does not recruit effective neural firing (Hutcheon and Yarom, 2000). Cao et al. (2018) demonstrated that neurons exhibit TI stimulation in a single neuron computational model. Esmaeilpour et al. (2021) investigated that the spatial selectivity of TI stimulation in deep brain areas depends on the phasic modulation of neural oscillations. TI stimulation can modulate spiking activity and facilitate phase synchronization, similar to transcranial alternating current stimulation (tACS; Howell and McIntyre, 2021). At similar field intensities, TI stimulation has less potent modulatory effects than other conventional TES (Negahbani et al., 2018). Mirzakhalili et al. (2020) found that TI stimulation requires a signal rectification process mediated by ion channels. The subthreshold neuromodulation of TI stimulation may be the most important effect (Chakraborty et al., 2018), and polarization effects can alter neural firing and synaptic transmission. Moreover, the potential mechanisms of TI stimulation may involve neurons, glial cells, and cerebral blood flow (Wachter et al., 2011; Monai et al., 2016). More studies are needed to explore and clarify the possible mechanisms.

### Development and Applications of TI Stimulation

Grossman et al. (2017) proposed that TI stimulation is noninvasive deep brain stimulation and carried out a series of experiments to validate the approach (Bouthour et al., 2017). Mouse neurons were fired with the Δf envelopes of electric fields with a patch clamp electrophysiological recording technique. To assess the focality and depth of TI stimulation, they applied 10 Hz of transcranial stimulation and 2,000 Hz + 2,010 Hz TI stimulation to the hippocampi of anesthetized mice and then measured the expression of the immediate early gene *c-fos* (an indicator of activated neurons). Transcranial stimulation at 10 Hz results in widespread expression in the cortex and hippocampus. By contrast, 2,000 Hz + 2,010 Hz of TI stimulation activates hippocampus regions without activating the cortex. They also explored behavioral responses to TI stimulation and found that TI stimulation can evoke forepaw movement. Stimulation regions can be altered by changing the ratios of currents without electrode movement. No pathophysiological activities and neural damage were observed (Grossman et al., 2017). Using the finite element method, Lee et al. (2020) found that optimized TI stimulation can successfully reach the hippocampus while reducing the effect of neocortical regions. Another study designed a TI stimulation method and validated its steerability through finite element analysis by using an action potential model and measuring waveforms in a saline solution (Xiao et al., 2019). The simulations of TI stimulation in a mouse head model achieved field strength in deep brain areas but less field strength in superficial areas (Grossman et al., 2017). Furthermore, the field strengths in a human model were much lower, and no direct stimulation effects were found; current higher than that in a mouse model might be required (Rampersad et al., 2019). Computational results indicated that the activation threshold increased with frequency and the envelope frequency had no association with the threshold. Moreover, the current intensity ratio altered the position of responding neurons. The characteristics of an envelope may predict the regions of TI stimulation (Gomez-Tames et al., 2021). Multichannel array electrodes for TI stimulation enhance focality and reduce scalp sensation in computational modeling and mouse experiments (Song et al., 2020). Wang H. et al. (2020) fabricated a TI stimulator that measures bioimpedance in real time and proposed an approach that can easily locate the target position. Current investigations on TI stimulation mainly use computational simulations and small animal experiments. A study investigated the variability in the electric field during TI stimulation and compared it to tACS. The results showed that the electric fields of TI stimulation are variable and more focal than those of tACS (von Conta et al., 2021). Hence, experiments on human subjects are necessary. Further human investigations on TI stimulation needs to be validated.

### Strengths and Challenges of TI Stimulation

In summary, the prospects of TI stimulation using noninvasive techniques are exciting. Conventional noninvasive TES usually generates scalp pain when exposing stimulation and limits the intensity of injection currents (Wu et al., 2021). TI stimulation can selectively stimulate specific brain regions, such as cortical and subcortical areas, thus preventing the stimulation of scalp nerves and scalp pain (Gomez-Tames et al., 2021). Given that DBS has remarkable therapeutic benefits for the treatment of Parkinson’s disease, tremor, and dystonia (Kringelbach et al., 2007), TI stimulation as a noninvasive DBS offers exciting prospects for the treatment of various brain disorders.

Most TI stimulation studies focus on computation and animal models, and thus human trials need to be further investigated. Given that anatomical differences affect electric field distributions, optimal TI stimulation parameters need to be further validated using various models. Furthermore, a specific positioning scheme for target regions is currently unavailable. An optimization algorithm focusing on the electric field in a target region should be established. Electrode fixation and interference location calculation should be accurate and convenient to facilitate clinical translation (Gunduz and Okun, 2017). TI stimulation currently cannot match the spatial resolution of implantable DBS. Deep small brain structures may not be specifically stimulated, such as the subthalamic nucleus (Grossman, 2018). Further studies are necessary to elucidate the related mechanism, which may involve neurons, synaptic plasticity, cerebral blood flow, and glial cells (Mirzakhalili et al., 2020). More importantly, the safety of TI stimulation needs to be examined and monitored. Finally, the prospect of TI stimulation neuromodulation method is highly promising, but the method requires further research before it can be applied to clinical processes.

## Near-Infrared Optogenetic Stimulation

**Figure 3:**
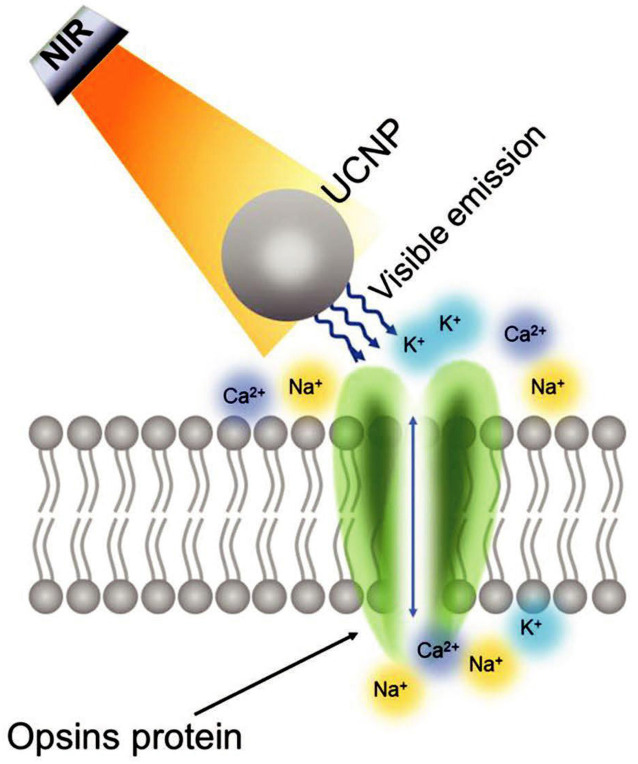
Near-infrared optogenetic stimulation. Schematic principle of lanthanide-doped upconversion nanoparticle (UCNP) mediated NIR optogenetic stimulation (Copyright permission was obtained from the publisher; Yu et al., 2019).

Near-infrared (NIR) optogenetic stimulation is a mode of optogenetic stimulation that does not require optical fiber implantation and has minimal invasiveness. Optogenetics is widely used in exploring neural circuits that govern sensory, memory, and motor behavior (Hausser, 2014). However, optogenetics requires the insertion of invasive optical fibers to target areas because the penetration depth of visible light is shallow. Blue-green wavelength penetration is limited because of the scattering and absorption by endogenous chromophores (Lin et al., 2013). NIR light (650–1,350 nm), which is much less scattered, can easily penetrate tissues and reach deep brain areas (Shi et al., 2016). NIR light-based photoregulation strategies offer means to modulate specific cells in deep brain structures (Chen G. et al., 2020). However, NIR light cannot be used directly and requires special processing. NIR optogenetic approaches stimulate deep brain regions by activating channel rhodopsin-expressing neurons, where NIR light is converted to visible light (Figure 3). By using this approach, researchers can control behavioral patterns simply by NIR illumination without performing optical fiber implantation (Chen et al., 2018). This approach shows great potential in bioimaging and neuromodulation because of its low imaging background and deep penetration (Wu et al., 2015).

### Mechanisms of NIR Optogenetic Stimulation

NIR optogenetic stimulation needs NIR light nanotransducers to exert its function. Typically, lanthanide-doped upconversion nanoparticles (UCNPs) can be modulated to a particular wavelength because of the special ladder-like electronic energy structures of trivalent lanthanide ions (Zhou et al., 2015). Dopant–host combination can control the emission wavelengths of UCNPs (Wang and Liu, 2009). The different site symmetries of dopant ions affect emission wavelength and emission peak intensity. The output color of UCNPs can be adjusted into a specific wavelength for optogenetic stimulation (All et al., 2019). UCNPs can convert low-energy NIR light to high-energy visible light (Prodi et al., 2015). UCNPs can be implanted close to optogenetic opsin neurons, and NIR illumination can be converted into visible light, which in turn activates optogenetic opsins, regulates light-gated ion channels that control the, and outflow of ions, and induces cell activation or suppression (Yu et al., 2019). Moreover, blended UCNPs with distinct excitation and emission wavelengths may result in neuron excitation and inhibition simultaneously within one region or multiple deep brain areas (Chen, 2019).

### Development and Applications of NIR Optogenetic Stimulation

UCNP-mediated optogenetics was first proposed by Deisseroth in 2011 (Chen S. et al., 2020). After a decade of research and development, NIR optogenetic stimulation has been investigated using different models (Ai et al., 2017; Wang et al., 2017; Ding et al., 2018). Hososhima et al. (2015) first used cultured cells containing UCNPs to observe the photoreactive responses that express channelrhodopsin. Neurons are triggered by NIR laser irradiation and generate action potential (Hososhima et al., 2015). Wu et al. (2016) synergized two unconversion booster effectors (dye-sensitizing and core/shell enhancement) to enhance upconversion efficiency; they successfully altered optogenetic neuron excitation to a biocompatible, water-solubilized, and deep-tissue penetrable 800 nm wavelength. The first *in vivo* study was investigated with *Caenorhabditis elegans* (*C. elegans*). *C. elegans* is widely used in optogenetic manipulation because of its small nervous system and quantifiable motor behavior (Zhen and Samuel, 2015). Bansal et al. (2016) implemented NIR optogenetic stimulation in *C. elegans* and found that it can activate channelrhodopsin-2 ion channels in mechanosensory neurons at a low average power with a quasi-continuous wave excitation approach. Further study showed that UCNPs can effectively activate inhibitory GABAergic motor neurons and excitatory glutamatergic DVC interneurons, leading to locomotion inhibition and activation (Ao et al., 2019). A recent study on zebrafish showed that NIR optogenetic stimulation can remotely activate channelrhodopsin-2 ion channels and effectively manipulate cation influx. This investigation provided a site-specific approach for regulating membrane-associated activities (Ai et al., 2017). More importantly, NIR optogenetic stimulation on live rodent animals was conducted in a number of experimental studies. Lin et al. (2017) packaged UCNPs in glass micropipettes and positioned them close to specific brain structures, such as the tegmental area, cortical striatum, and visual cortex. The results showed that NIR light remotely activated targeting brain structures and showed great biocompatibility (Lin et al., 2017). They then implanted a microscale upconversion-based device into a mouse brain and successfully controlled motor function in awake and freely moving animal (Wang et al., 2017; Lin et al., 2018). NIR optogenetic stimulation represented a major leap when Chen et al. published their findings in the Science journal (Feliu et al., 2018). They demonstrated that UCNP-mediated NIR approach regulated multiple neuronal activities in deep brain regions, specifically evoking dopamine release in the ventral tegmental area, inducing brain oscillations by activating the medial septum, and silencing seizure by inhibiting hippocampal cells and triggering memory recall. In addition, the study showed the excellent biocompatibility, flexibility, robust minimal invasiveness, long-term *in vivo* utility, low dispersion, and negligible cytotoxicity of the approach (Chen et al., 2018). One large timescale study demonstrated that NIR optogenetic stimulation successfully controlled animal locomotive behavior by manipulating neurons in the dorsal striatum and UCNPs remained functional for at least 8 weeks at the injection brain site; these results suggested that using this approach in long-term behavioral tests is highly feasible (Miyazaki et al., 2019). Ma et al. (2019) reported that injected UCNPs enable retinal photoreceptors to perceive NIR light and differentiate sophisticated NIR shape patterns. This type of vision is compatible with daylight vision, offering options for mammalian vision repair and enhancement (Ma et al., 2019). Liu et al. (2021) developed NIR multicolor optogenetics using trichromatic UCNPs, which can selectively activate three distinct neuronal populations and modulate motor behavior in awake mice.

### Strengths and Challenges of NIR Optogenetic Stimulation

NIR optogenetic stimulation offers the possibility of delivering light to deep brain regions, is less invasiveness, and has a high spatial resolution and cell specificity (Chen S. et al., 2020). Compared with optogenetics, NIR optogenetic stimulation can be manipulated remotely in the brain without resulting in behavior restriction (All et al., 2019; Lin et al., 2021). This approach has been validated *in vitro* and *in vivo* in terms of its capability to modulate neural activities, and the results suggested potential neuroscience applications. Safety has been demonstrated in many studies, as well as good biocompatibility and negligible toxicity. Furthermore, advancements in NIR optogenetic stimulation require collaboration among physicists, chemists, neuroscientists, and biologists. It is a big step toward the remote and noninvasive control of brain activities. Hence, transferring this approach to clinical trials is possible, and it may complement current neurological disorder therapies, such as DBS.

Some challenges encountered in NIR optogenetic stimulation need to be addressed here. The toxicity of UCNPs on the cellular, tissue, and organ levels should be comprehensively investigated in order that potential organ damage can be prevented, and long-term biocompatibility studies should be conducted given that UCNPs may change properties and are readily endocytosed by cells (Nazarenus et al., 2014). Effective nanostructures should be designed to satisfy different studies (Tao et al., 2020). In addition, further investigations using large animals are required before clinical trials. Despite such challenges, the recent discovery of NIR optogenetic stimulation is a significant breakthrough. We believe that this new technology has bright therapeutic prospects.

## Nanomaterial-Enabled Magnetic Stimulation

Magnetic fields can penetrate tissues with less attenuation and harmless effects, thus having potential uses in wireless and noninvasive methods for modulating brain activities (Christiansen et al., 2019). Magnetic fields are considered intermediary and should be converted to localized secondary stimuli (Wang and Guo, 2016). Methods combining magnetic fields with magnetic nanoparticles (MNPs) converting magnetic signals have been investigated with different techniques (Huang et al., 2010; Wang G. et al., 2020; Kozielski et al., 2021). MNPs as transducers can be categorized into magnetothermal activation, magnetoelectric activation, and magnetomechanical activation (Roet et al., 2019). MNPs incorporate ion-transporting proteins, which can be transgenically expressed in neurons and respond to changes in heat, electricity, or force (Christiansen et al., 2019). It is commonly known as nanomaterial-enabled magnetic stimulation. This approach represents a more effective stimulation that can noninvasively modulate deep brain neural activities and selectively activate specific neural circuits.

### Mechanisms of Nanomaterial-Enabled Magnetic Stimulation

**Figure 4:**
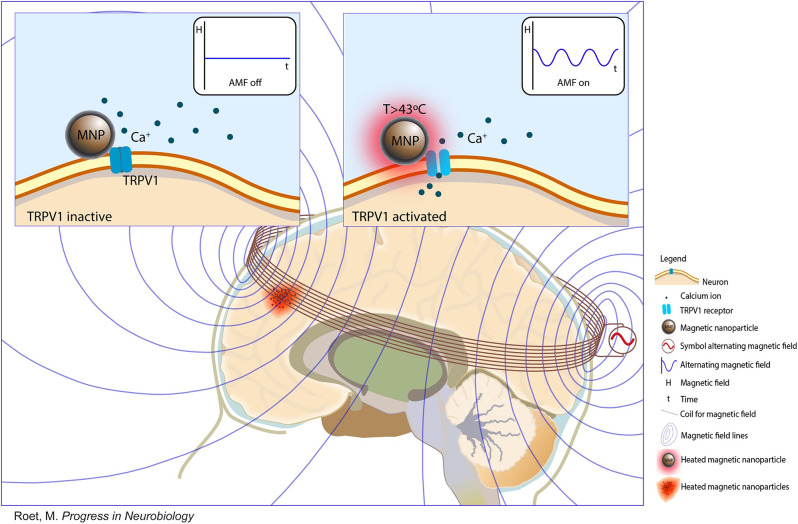
Nanomaterial-enabled magnetic stimulation. A schematic view and schematic principle of nanomaterial-enabled magnetic stimulation in the human brain (Copyright permission was obtained from the publisher; Roet et al., 2019).

Magnetothermal activation uses alternating magnetic fields (AMFs) to activate the temperature-gated ion channels of transient receptor potential vanilloid (TRPV) family (Figure 4). MNPs can fuse to TRPV and generate heat through hysteretic power loss and then induce calcium ion influx, membrane depolarization, and action potential firing (Huang et al., 2010; Munshi et al., 2017). TRPV1 is endogenously expressed in mammalian neurons (Starowicz et al., 2008; Terzian et al., 2014). Some studies used genetic tools to achieve the uniform expression of TRPV1 in specific brain areas in mice (Huang et al., 2010; Temel and Jahanshahi, 2015). Magnetoelectric activation uses magnetoelectric nanoparticles (MENs) to generate local electric fields under an external magnetic field. The electric field originates from the intrinsic coupling between electric and magnetic fields in MENs (Guduru et al., 2015). Magnetomechanical activation uses MNPs to convert the energy of magnetic fields into mechanical forces (Chen M. et al., 2020). These forces can activate pressure-sensitive receptors and subsequently modulate neurons (Shin and Cheon, 2017).

**Table 1:** Overview of each type of neuromodulation.

	FUS	TI stimulation	NIR optogenetic stimulation	Nanomaterial-enabled magnetic stimulation
Energy delivery	Ultrasound	Electrical	Near-infrared	Magnetic
Invasiveness	Noninvasive	Noninvasive	Minimally invasive	Minimally invasive
Spatial resolution	~1 mm	> mm	<1 mm	<1 mm
Depth of penetration	10–15 cm or more	5 cm or more	1 cm or more	Unlimited in theory
Gene delivery	No	No	Yes	Yes
Experiment animal models	Rodents, non-human primates, human	Rodents, human	Rodents	Rodents
Stimulation mode	Fixing transducer	Fixing electrodes	Remote	Remote
Complexity level	Moderate	Moderate	Complicated	Complicated
Reversible	Yes	Yes	No	No
Cost	Moderate	Low	High	High

*FUS, focused ultrasound*.

### Development and Applications of Nanomaterial-Enabled Magnetic Stimulation

Huang et al. (2010) first demonstrated that superparamagnetic nanoparticles exposed to AMFs can locally generate heat and remotely activate TRPV1, eliciting responses from human embryonic kidney 293 cells and *C. elegans*. Another study showed that modified TRPV1 with MNPs can modulate calcium influx *in vivo* and *in vitro* when exposed to a magnetic field (Stanley et al., 2012). Further studies aimed to determine whether a magnetic field can regulate the behavior of rodents animals. Radio wave or magnetic field treatment for glucokinase–Cre (GK–Cre) mice that received ventromedial hypothalamus injection of Ad-FLEX-anti-GFP-TRPV1/GFP-ferritin alters blood glucose and food intake (Stanley et al., 2016). However, the above investigations did not discuss the mechanisms of neural modulation. Chen et al. (2015) exerted a considerable amount of effort into studying wireless magnetothermal activation. In mice, the hysteretic heating of MNPs activates hippocampal and ventral tegmental area neurons after the application of AMFs. To ensure the sustained and uniform levels of TRPV1 expression, the author designed a transgene across a cell membrane. Meanwhile, magnetothermal deep brain stimulation has minimal cytotoxicity, long-term, biocompatibility, and stability (Chen et al., 2015). Munshi et al. (2017) first reported magnetothermal activation using MNPs in awake and freely moving animals. Magnetothermal stimulation in the motor cortex or striatum evokes different types of motor behavior, and the duration of behavior correlates with magnetic field application (Munshi et al., 2017). In addition, they transfected rat hippocampal neurons to express thermosensitive chloride channel anoctamin 1 and silenced neuronal activity by applying a magnetic field to target neurons (Munshi et al., 2018). The behavioral responses evoked by magnetothermal activation result from optogenetic or chemogenetic neural modulation. Hescham et al. (2021) used a wireless magnetothermal approach for parkinsonian-like mice. The results revealed that magnetothermal neuromodulation in the STN can not only modulate the motor behavior of healthy mice remotely but also reverse motor deficits (Hescham et al., 2021). MNPs offer attractive methods for brain tumor therapies because magnetic fields can stimulate tumors through heating without damaging healthy hypodermal tissues (Thorat et al., 2016, 2019). This approach prevents the serious adverse effects of traditional chemotherapy. Compared with magnetothermal activation, magnetoelectric activation, and magnetomechanical activation have not been extensively explored. Nguyen et al. (2021) intravenously injected MENs and forced them to cross the BBB and localize to the cortical areas by using a magnetic field gradient. The results showed that cortical neurons and cortical networks can be activated by an external magnetic field (Nguyen et al., 2021). Kozielski et al. (2021) demonstrated that the magnetic stimulation of MENs can modulate neuronal activities in the motor cortex and nonmotor thalamus and modulate mice behavior. Overall, nanomaterial-enabled magnetic stimulation may facilitate remote noninvasive deep brain stimulation without genetic manipulation.

### Strengths and Challenges of Nanomaterial-Enabled Magnetic Stimulation

Nanomaterial-enabled magnetic stimulation has offered broad application prospects for noninvasive deep brain modulation. The approach provides high spatial resolution and cell specificity. Its feasibility, effectiveness, biocompatibility, stability, and safety have been validated *in vitro* and *in vivo* (Chen et al., 2015; Park et al., 2020; Wang G. et al., 2020; Kozielski et al., 2021). More importantly, nanomaterial-enabled magnetic stimulation can utilize MNPs for the modulation of neurons with heat or electric or mechanical forces without genetically engineering. This feature is important and may ensure clinical trial approval (Starowicz et al., 2008). The chemical composition of MNPs is similar to that of MRI agents by having minimal cytotoxicity and long-term effectiveness (Petters et al., 2014; Roet et al., 2019). Meanwhile, with the development of nanotechnology, MNPs have huge biomedical application potential (Chen et al., 2017; Manescu Paltanea et al., 2021).

Studies on nanomaterial-enabled magnetic stimulation mainly focused on small animal models. The next step should be conducting studies on non-human primates and even clinical trials. Scaling AMF coils to human deep brain areas is a huge challenge. In addition, The heating side effects of MNPs should be considered because they may result in brain swelling and increase intracranial pressure (Maier-Hauff et al., 2007). Moreover, heating can promote MNP aggregation, which may cause occlusion in the blood vessel (Wegscheid et al., 2014) and ultimately lead to serious clinical consequences. Therefore, solving this problem is highly necessary. Lastly, the long-term toxicological effects and clearance of MNPs in the brain regions should be investigated.

## Future Trends

The above deep brain neuromodulation techniques mainly rely on acoustic, electronic, optical, and magnetic signals and show great promise as a high-spatiotemporal resolution and deep penetration platform. These approaches are noninvasive or minimally invasive. The characteristics of the four types of neuromodulation are summarized in Table 1.

FUS and TI stimulation are noninvasive neuromodulations without gene delivery. It is relatively easy to translate to the clinic. These techniques may serve as complementary neuromodulation for the treatment of brain disorders. We believe that FUS and TI could be an upgrade of traditional DBS to improve efficiency and safety. However, a similar situation as DBS, FUS, and TI stimulation may just alleviate the progression but cannot cure the disease. Furthermore, none of these approaches has cell type-specific to the brain target. Therefore, future research should explore the underlying mechanisms behind FUS and TI stimulation so that the results can be optimized for clinical application.

Compared to FUS and TI stimulation, NIR optogenetic stimulation and nanomaterial-enabled magnetic stimulation have a long way to go for clinical application. Both approaches need gene delivery. So there are a number of practical challenges before clinical application. First, the long-term safety of viral vectors used for genetic modification to the target neurons has yet to be fully illustrated. Second, maintaining the gene delivery effective and stable in different animal models especially non-human primates is also a potential challenge. Third, genetic therapy for primates is much more complicated, and high cost and long cycle are required for the research. Therefore, there remains much to be done before NIR optogenetic stimulation and nanomaterial-enabled magnetic stimulation can be delivered to the clinical arena. Promisingly, gene therapy has been increasingly applied to treat tumors, virus infection, and genetic disease. As gene delivery technologies develop, the application will be continuously updated. That would be of great significance in neuromodulation.

## Conclusions

These techniques may represent next-generation neural interface tools for neuroscience and have huge potential as tools for advancing neuroscience research. Cross-disciplinary collaboration is needed to establish an optimal scheme given and confirm that these techniques are indeed next-generation noninvasive DBS technologies. We believe that advancements in these techniques will pave the way for novel therapeutic options for brain disorders.

## Author Contributions

XL and FQ wrote the manuscript. LH performed revision and improved the quality of the manuscript. XW edited and revised the manuscript. All authors contributed to the article and approved the submitted version.

## Funding

This work was supported by a grant from the key program of National Natural Science Foundation of China (No. 11932013).

## Conflict of Interest

The authors declare that the research was conducted in the absence of any commercial or financial relationships that could be construed as a potential conflict of interest.

## Publisher’s Note

All claims expressed in this article are solely those of the authors and do not necessarily represent those of their affiliated organizations, or those of the publisher, the editors and the reviewers. Any product that may be evaluated in this article, or claim that may be made by its manufacturer, is not guaranteed or endorsed by the publisher.
